# O01 The consumption of human antibiotics in veterinary medicine, in Estonia, 2006–22

**DOI:** 10.1093/jacamr/dlad066.001

**Published:** 2023-06-14

**Authors:** Marju Sammul

**Affiliations:** Estonian University of Life Sciences, Estonia; State Agency of Medicines, Estonia

## Abstract

**Objectives:**

In the absence of authorized veterinary medicinal products, veterinarians can use authorized human medicines, if necessary. The number of registered veterinary medicinal products, including veterinary antibiotics, has increased, over the years. Reducing the use of medically important antimicrobials in veterinary medicine is increasingly important to avoid the development and spread of antimicrobial resistance. The aim of this study was to analyse 17 year trends in the use of human antibiotics in veterinary medicine, in Estonia.

**Methods:**

The data of human antibiotics used in veterinary medicine are based on wholesaler’s reports collected by the Estonian State Agency of Medicines, in 2006–2022. The sales data include total sales of human antimicrobial medicinal products to veterinarians and are collected at package level per year, including the name of the medicinal product, identification number of the product, active substance, pharmaceutical form, strength, package size and number of sold packages.

**Results:**

Between 2006 and 2022, the overall sales of human antibiotics to veterinarians increased from 40.6 kg in 2006 to 54.4 kg in 2022 (peak 71.6 kg in 2019). In Estonia, based on 17 years of data, 220 different human antimicrobial medicinal products were sold to veterinarians, an average 91 different products per year (range 79–101). The number of different human antibiotic substances used in veterinary medicine was 45 ± 2 per year, 60 in total. The most used antibiotics over the 17 years were orally administered amoxicillin, clindamycin, ciprofloxacin, sulfamethoxazole and doxycycline and injectable amoxicillin and cefazolin. The turnover of human antibiotics sold to veterinarians increased from 34 000 EUR in 2006 to 99 000 EUR in 2022. Over the years, analysed by the product form, the most sold human antibiotics for veterinary use were products for oral administration, accounted for 90% of total quantity of antibiotics (24 different substances), followed by injections (9%, 19 different substances) and dermatological and sensory preparations (0.6%, 7 and 9 different substances, respectively). From medically important antibiotics, meropenem, imipenem, piperacillin, ceftriaxone, vancomycin and mupirocin were used in small quantities throughout the years.

**Conclusions:**

Despite the number of registered veterinary antibiotics having increased over the years, the consumption of human antibiotics used in veterinary medicine has increased by the total quantity and by the turnover. The number of human antibiotic substances and sold packages has no significant changes.

